# Mesoscopic Mapping of Visual Pathway in a Female 5XFAD Mouse Model of Alzheimer’s Disease

**DOI:** 10.3390/cells11233901

**Published:** 2022-12-02

**Authors:** Yunkwon Nam, Sujin Kim, Jieun Kim, Hyang-Sook Hoe, Minho Moon

**Affiliations:** 1 Department of Biochemistry, College of Medicine, Konyang University, Daejeon 35365, Republic of Korea; 2 Research Institute for Dementia Science, Konyang University, Daejeon 35365, Republic of Korea; 3 Department of Neural Development and Disease, Korea Brain Research Institute (KBRI), Daegu 41068, Republic of Korea; 4 Department of Brain & Cognitive Sciences, Daegu Gyeongbuk Institute of Science & Technology (DGIST), Daegu 42988, Republic of Korea

**Keywords:** Alzheimer’s disease, visual pathway, 5XFAD mice, cholera toxin β subunits

## Abstract

Amyloid-β (Aβ) deposition and Aβ-induced neurodegeneration appear in the retina and retinorecipient areas in the early stages of Alzheimer’s disease (AD). Although these Aβ-related changes in the retina cause damage to the visual functions, no studies have yet revealed the alterations in the visual pathways of AD. Therefore, we investigated the alterations of visual circuits in the AD mouse model using anterograde tracer cholera toxin β subunits (CTβ). Moreover, we investigated the Aβ accumulation in the retina and retinorecipient areas and the neuronal loss, and synaptic degeneration in retinorecipient areas by immunofluorescent staining of 4- and 12-month-old female 5XFAD transgenic mice. Our results demonstrated that Aβ accumulation and neurodegeneration occurred in the retina and retinorecipient regions of early and late stages of the 5XFAD mice. Retinal efferents to the suprachiasmatic nucleus and lateral geniculate nucleus were impaired in the early stage of AD. Moreover, retinal connections to the dorsal lateral geniculate nucleus and superior colliculus were degenerated in the late-stage of AD. These findings reveal the Aβ-related pathology induced visual circuit disturbances at the mesoscale level in both the early and late stages of AD and provide anatomical and functional insights into the visual circuitry of AD.

## 1. Introduction

Amyloid-β (Aβ) peptides, one of the causative factors in Alzheimer’s disease (AD), are natural products of metabolism consisting of 36–43 amino acids. Under pathological conditions, the production functions of Aβ increase, and the clearance functions decrease. An imbalance between the production and elimination functions of these Aβ increases the aggregation and accumulation of Aβ [1]. Notably, the intermediate and soluble oligomer Aβ forms provoke neurodegeneration including neuronal death and synaptic loss in the brain [2]. Interestingly, a study in which Aβ was injected into neurons using a patch pipette reported that intracellular accumulation of Aβ induces synaptic dysfunction [3]. The neurotoxic and synaptotoxic Aβ directly attenuates the neural circuit networks in the brains affected by AD [4,5,6,7]. A decrease in structural and functional connectivity of neural circuit networks directly affects the onset and progression of AD symptoms [8,9]. Impairments in various neural circuits and brain networks that underlie cognitive, behavioral, and sensory systems could result in cognitive decline, emotional deficit, and sensory loss in AD [10]. Consequently, circuit disturbances and network disruptions resulting from Aβ toxicity directly induce the accompanying dysfunctions of various systems in AD. Therefore, it is essential to know which circuits and networks are altered and damaged in AD.

Visual impairments, such as low visual acuity and reduced contrast sensitivity, are common in patients with AD [11]. Many studies have reported that retinal ganglion cells (RGCs), particularly intrinsically proprioceptive retinal ganglion cells (ipRGCs), and the retinal nerve fiber layer (RNFL) are severely damaged in AD [12,13,14]. These RGCs and RNFL undergo extensive damage in the early stage of AD without visual failure [13,15]. It suggests that pathophysiologic alterations have already appeared in the eyes of the early stage of AD. Indeed, Aβ accumulation and neuronal loss were significantly increased in the retina of AD patients compared to healthy controls [16]. Similar to the results of the AD patients, Aβ accumulation in the retina precedes Aβ deposition in AD mouse models [17,18]. Moreover, damaged inner retina induced by Aβ aggregation precedes neuronal cell death in other brain regions [18]. Taken together, Aβ-induced deficiency of RGCs and RNFLs appears from the early stages of AD, and this deficiency can lead to impaired connectivity between the retina and retinorecipient areas. Therefore, alterations in the visual pathway from the eye to the brain could affect visual dysfunction in AD.

In the brain, retinorecipient areas originating from the eye include 25 ipsilateral brain regions and 34 contralateral brain regions [19]. The major retinorecipient areas are the lateral geniculate nucleus (LGN), superior colliculus (SC), suprachiasmatic nucleus (SCN), olivary pretectal nucleus (OPN), and medial terminal nucleus (MTN) of the accessory optic tract [20]. The LGN is divided into subregions, such as the dorsal lateral geniculate nucleus (dLGN), intergeniculate leaflet (IGL), and ventral lateral geniculate nucleus (vLGN) [21]. Although the connectivity between retina and retinorecipient regions in the healthy brain is well known, no studies the alterations of the visual pathways in the brains with AD. Moreover, it has been shown that Aβ-induced deficiency of RGC and RNFL appears in the early stages of AD, resulting in visual pathway impairment. Therefore, a hypothesis was formulated stating that the visual pathway could be altered in Aβ-overexpressing transgenic mice before cognitive decline as the primary symptom of AD. Importantly, visual behavioral deficits in AD patients are known to occur at the prodromal stage of AD [10]. Likewise, it has been reported that visual-related behavioral deficits, such as circadian rhythm (sleep-wake) disturbance and visual acuity (optokinetic system) impairment, appear in 5XFAD mice between 4 and 6 months of age [22,23]. Herein, we focused on the topographical changes in visual pathways before and after cognitive impairment. We selected 4-month-old 5XFAD mice corresponding to prodromal stage of AD [24] and 12-month-old 5XFAD mice corresponding to severe stage of AD [25]. To reveal the connections from the retina to retinorecipient areas in AD, we used the cholera toxin β subunit (CTβ) conjugated to Alexa-Fluor 488 as a tracer. CTβ is a non-transsynaptic neural tracer widely used for monosynaptic mapping since it lacks transsynaptic tracing [26]. In addition, CTβ is a reliable tracer widely used to evaluate the anterograde transport of RGCs [17,27,28].

## 2. Materials and Methods

### 2.1. Animals

The five familial AD mutations (5XFAD) mice express three mutations in the human *APP* gene (Florida I716V, Swedish K670N/M671L, and London V717I) and two mutations in the human *PSEN1* gene (L286V and M146L). The animals were purchased from the Jackson Laboratory in Bar Harbor, Mainer, ME, USA (#006554). The present study used 4- and 12-month-old female 5XFAD mice and wild-type (WT) littermates. The 5XFAD mice are Aβ-overexpressing transgenic mice with five familial AD mutations, generally showing significant cognitive impairment from 6 months of age and neurodegeneration from 9 months of age [29,30]. As previously described [31], the 5XFAD transgenic mice were identified through genotyping of tail DNA. Moreover, some 5XFAD and WT littermate mice start to exhibit retinal degeneration and lose sight at 35 days of age as a result of phosphodiesterase-6b retinal degeneration-1 (*Pde6b^rd1^*) allele in the SJL/J background strain [32]. *Pde6b^rd1^* has an autosomal recessive inheritance pattern [33]. Thus, mice with retinal degeneration *Pde6b^rd1^* mutation were classified and excluded using genotyping (Mutant: 5-AAG CTA GCT GCA GTA ACG CCA TTT-3, Wild-type: 5-ACC TGC ATG TGA ACC CAG TAT TCT ATC-3, Common: 5-CTA CAG CCC CTC TCC AAG GTT TAT AG-3). WT and 5XFAD mice excluding *Pde6b^rd1^* mutants were randomly assigned into four groups: (1) 4-month-old WT mice (n = 5), (2) 4-month-old 5XFAD mice (n = 5), (3) 12-month-old WT mice (n = 5), and (4) 12-month-old 5XFAD mice (n = 5). Maintenance and treatment of the animals were performed as per the principles of the Care and Use of Laboratory Animals (NIH Publication No. 85-23, revised 1985) and the Animal Care and Use Guidelines of Konyang University (Project code: P-18-06-A-01). Every attempt was made to keep the animals alive and their suffering to a minimum.

### 2.2. Intravitreal Injections

In a mouse, 85–90% of optic nerve axons decussate to the other side of the brain [34]. In addition, it has been reported that axons arising from the nasal retina project through the contralateral optic tract to the contralateral brain [35]. Thus, we injected CTβ intravitreally towards the nasal retina. All the animals were anesthetized with a dose of 250 μg/kg Avertin (Tribromoethanol; Sigma-Aldrich, St. Louis, MO, USA). The intravitreal injection was performed using a microscope (Leica, stereozoom S9 D). A sharp 30-gauge needle was inserted into the edge of the right eye to make a small incision and 1 µL of vitreous fluid was subsequently withdrawn using a blunt 33-gauge needle attached to a Hamilton syringe. Finally, 1 µL of 1 mg/mL CTβ conjugated to Alexa-Fluor 488 (Thermo Fisher Scientific Inc., Waltham, MA, USA) was injected into the right eye using the same Hamilton syringe [17]. In order to prevent eye damage, a needle was placed between the lens and the retina, and eye ointment was applied following the intravitreal injection.

### 2.3. Brain Tissue and Retina Wholemount Preparation

We analyzed the mouse’s left brain, contralateral to the right eye. The animals were anesthetized for three days following the CTβ injections, transcardially perfused with 0.05 M phosphate-buffered saline (PBS), and subsequently fixed with cold 4% paraformaldehyde in 0.1 M phosphate buffer (PB). Thereafter, the eyes and brain tissues were extracted, and the tissues were post-fixed in 0.1 M PB containing 4% paraformaldehyde for 20 h at 4 °C. For cryoprotection, the brains were subsequently immersed in 30% sucrose in 0.05 M PBS solution for 3 days at 4 °C. The brains were subsequently embedded in Surgipath^®^ frozen section compound (Leica Biosystems, Wetzlar, Germany) and sliced into serial 30-μm-thick coronal slices using a CM1850 cryostat (Leica Biosystems). As previously described [36], for the retina wholemount preparation, the eye tissues were dissected to remove the lens, vitreous, and retinal pigment epithelium, and the retina was collected. Then, quadrants of the retina were cut and spread flat on the glass.

### 2.4. Immunofluorescence Labeling

To investigate the immunoreactivity of Aβ accumulation, the retina and brain tissues were incubated using a blocking solution (0.05% bovine serum albumin, 1.5% normal goat serum, and 0.3% Triton X-100 in PBS) and mouse anti-4G8 antibody specific for Aβ_17-24_ (1:2000; BioLegend, San Diego, CA, USA) overnight at 4 °C. To examine the immunoreactivity of neuronal nuclei (NeuN) and synaptophysin (SYN), free-floating brain sections were incubated overnight at 4 °C with the mouse anti-NeuN antibody (1:1000; Cat.# MAB377, Merk KGaA, Darmstadt, Germany) or mouse anti-SYN antibody (1:500; Cat.#S5768, Sigma-Aldrich), respectively, in blocking solution. Additionally, the retina and brain tissues were incubated with donkey Alexa 488-conjugated anti-mouse IgG (1:200; Thermo Fisher Scientific Inc., Waltham, MA, USA) or donkey Alexa 594-conjugated anti-mouse IgG (1:200; Thermo Fisher Scientific Inc.) for 1 h at room temperature. To counterstain the nuclei, the retina and brain tissues were mounted on ProbeOnTM Plus Microscope Slides (Thermo Fisher Scientific Inc.) and covered with Fluoroshield^TM^ with 4′,6-diamidino-2-phenylindole (DAPI) (Sigma-Aldrich).

### 2.5. Image Acquisition and Analysis

All the brain and retinal tissues labeled with CTβ and 4G8, NeuN, and SYN staining were imaged using a Zeiss LSM 700 Meta confocal microscope (Carl Zeiss AG, Ober-kochen, Germany) and analyzed using ImageJ software (National Institutes of Health, Bethesda, MD, USA). To quantify the percentage of 4G8 (+) area 20 to 40 images of the immunostained retina sections were quantified as follow (Supplementary Figures S1 and S2); (1) the images were changed to 8-bit images; (2) the images were thresholded to eliminate background signals; (3) sorting the 4G8 (+) labeled signals (>20 μm); (4) the thresholded images of the defined retina region were quantified by the “analyze particles” tool for the “% area” measurement of the 4G8 (+) labeled signals. To quantify the number NeuN (+) cells per mm^2^, 20 to 40 images of the immunostained brain sections were quantified as follows: (1) the positively-stained areas (10–20 μm) were manually outlined using the paintbrush tool; (2) the images were changed to 8-bit images; (3) the images were thresholded to eliminate background signals; (4) the anatomical retinorecipient regions of the SC, LGN, IGL, and SCN was defined based on DAPI; (5) the thresholded images of the defined brain region were quantified using the “analyze particles” tool and the “count” value was read; and (6) the “count” value was divided by the area of the defined region. Last, the methods of measurement of the optical density of SYN immunoreactivity and CTβ-labeled axon terminal were as follows: (1) manually removing excessively merged forms around the Aβ plaque; (2) manually drawing the region of interest (ROI); (3) selecting “analyze” and “measure” to give a numerical measurement to the fluorescence of the ROI. In particular, values of the CTβ-labeled axon terminal from the four groups were normalized to the controls using the following equations: % of control = (fluorescence intensity of WT or 5XFAD mice/fluorescence intensity average of WT mice) × 100.

### 2.6. Statistical Analysis

All the data were analyzed by GraphPad Prism 7 (Systat Software, La Jolla, CA, USA). Analyzed values are plotted as mean ± standard error of the mean (SEM). Data were analyzed using independent t-tests between the two groups and two-way analysis of variance (ANOVA) was performed between the four groups followed by Tukey’s post hoc tests. A *p*-value of less than 0.05 was considered statistically significant. Image acquisition, quantification, and statistical analysis of each group were blindly performed.

## 3. Results

### 3.1. Histological Profiling of the Aβ Accumulation and Retinal Projections to the Brain

**Figure 1:**
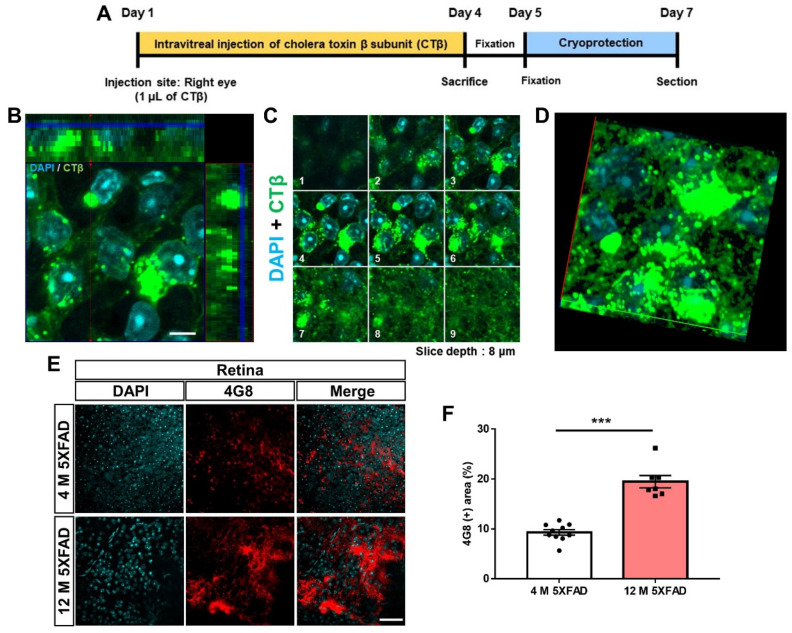
Validation of the application of cholera toxin β subunit (CTβ) and characterization of amyloid-β (Aβ) deposition in the retina. (**A**) The schematic diagram shows the overall experimental design. (**B**) An orthogonal view of the z-stack images indicates that the CTβ has an intracellular localization in retinal cells. The side and bottom panels, respectively, depict y–z and x–z cross-sectional images. Scale bar = 5 μm. (**C**) Serial z-stack images consist of 9 sections at 1-μm intervals. (**D**) The representative image shows a three-dimensional z-projection of the acquired stack. (**E**) The representative image shows Aβ accumulation in the retina by immunohistochemical staining with anti-4G8 antibody. Scale bar = 50 μm. (**F**) The quantitative graph exhibits the area fraction of Aβ in the retina of 5XFAD mice (7–10 images of the retina wholemount sections were obtained from 5 mice in each group). Statistical analysis was conducted by an independent *t*-test. *** *p* < 0.001 indicate a significant difference.

To investigate the retinorecipient areas and retinal pathways in the brain with AD, we injected CTβ, a non-transsynaptic anterograde trace, into the vitreous of WT and 5XFAD mice (Figure 1A). Three days following the intravitreal injection, the fluorescence of CTβ conjugated to Alexa-Fluor 488 was detected in the retina (Supplementary Figure S3). Next, we investigated the subcellular localization of CTβ in the retinal flat mount using Z-stack imaging. CTβ fluorescence signals were localized in the cytoplasm of retinal cells stained with DAPI (Figure 1B–D). This suggests that retinal cells present in the RGCs layer, a layer in which many cells are distributed, endocytosed CTβ. Moreover, to examine whether Aβ accumulations were deposited in the retinal RGCs, we immunofluorescence staining was conducted using anti-4G8 antibody of 4-month- and 12-month-old 5XFAD mice (Figure 1E). We found that Aβ peptides were significantly aggregated in the retina of 12-month-old 5XFAD compared to 4-month-old 5XFAD mice (Figure 1F). Aβ accumulates little in the retina of WT mice [22,37] (Supplementary Figure S4). These data suggest that Aβ accumulation increases with AD progression.

**Figure 2:**
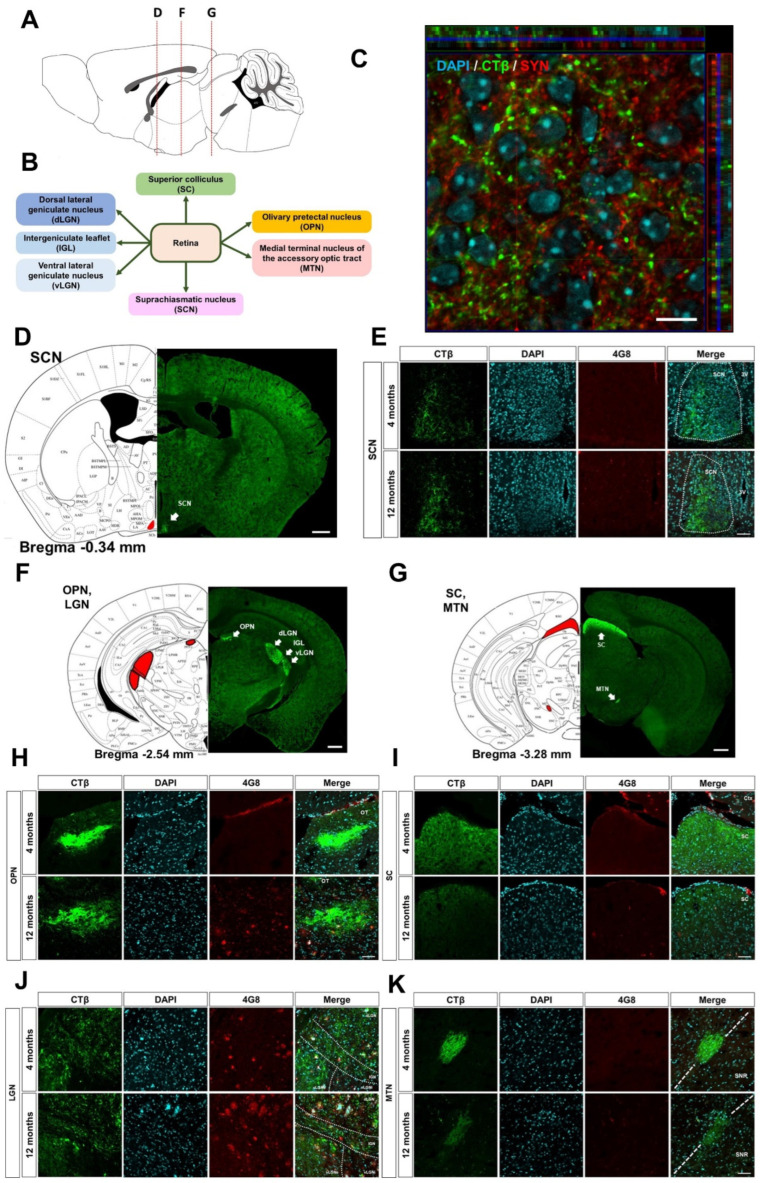
Histological profiling of the CTβ and Aβ deposition in the retinorecipient regions. (**A**) The sagittal plane of the mouse brain indicates the positions of the representative Figure 2 in (**D**,**F**,**G**). (**B**) The schematic diagram indicates the projection from the retina to the retinorecipient regions of the brain. CTβ-positive signals represent the retinal output to the (**D**) SCN, (**F**) OPN/LGN, and (**G**) SC/MTN in the brain hemisphere. Scale bar = 500 μm. (**C**) The representative orthogonal view shows that CTβ co-localizes with the presynaptic vesicular protein synaptophysin (SYN) in the SCN. Green indicated CTβ-positive signals. Red signals showed SYN-positive signals. The side and bottom panels, respectively, depict the y−z and x−z cross-sectional images. Scale bar = 10 μm. Representative images show the deposition of Aβ plaques stained with 4G8 antibody in the (**E**) SCN, (**H**) OPN, (**I**) SC, (**J**) LGN, and (**K**) MTN of 4- and 12-month-old 5XFAD groups. Scale bar = 50 μm. The dotted line in (**J**) indicates the sub-region of the LGN. LGN is divided into dLGN, IGL, external and internal vLGN. (**K**) MTN is located at the boundary of SNR. dLGN, dorsal lateral geniculate nucleus; IGL, intergeniculate leaflet; LGN, lateral geniculate nucleus; MTN, medial terminal nucleus of the accessory optic tract; OPN, olivary pretectal nucleus; OT, nucleus of the optic tract; SC, superior colliculus; SCN, suprachiasmatic nucleus; SNR, substantia nigra pars reticula; vLGN, ventral lateral geniculate nucleus.

Subsequently, we compared the retinal efferents to the brain regions of the healthy and AD animals (Figure 2A,B). CTβ was mainly co-localized with SYN (Figure 2C and Supplementary Figure S5). This result suggests that CTβ endocytosed into RGCs is transported to their axon terminals reaching the retinorecipient regions. As in previously reported studies [19,20], the signals of CTβ axonally transported from retina were detected in retinorecipient areas, including the SCN, OPN, LGN, SC, and MTN of WT and 5XFAD mice (Figure 2D,F,G). Moreover, we characterized Aβ deposition in retinorecipient areas of 4- and 12-month-old 5XFAD mice (Figure 2E,H,K). Aβ accumulation was not observed in the SCN, SC, and MTN of 4- and 12-month-old 5XFAD mice (Figure 2E,I,K). Interestingly, Aβ accumulation was not detected in the OPN of 4-month-old 5XFAD mice, whereas Aβ deposition was observed in 12-month-old 5XFAD mice (Figure 2H). The LGN showed Aβ deposition in both 4- and 12-month-old 5XFAD mice (Figure 2J).

### 3.2. Disruption of the Retina-SCN Connections in Animal Model of AD

**Figure 3:**
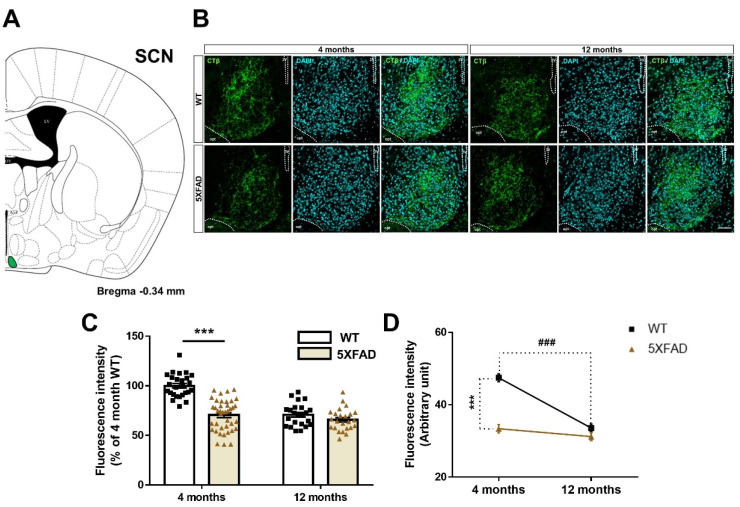
Impairment of the retina-suprachiasmatic nucleus (SCN) pathway in WT and 5XFAD mice. (**A**) Diagram of a mouse brain section explaining the location of the SCN. (**B**) The representative image shows CTβ-labeled terminal buttons of the axon in the SCN of 4- and 12-month-old WT and 5XFAD mice. Scale bar = 50 μm. (**C**) The quantitative graph exhibits the fluorescence intensity of the CTβ (+) area in the SCN of WT and 5XFAD mice (24–40 images of the brain sections were obtained from 5 mice in each group). (**D**) Comparison of the impairment of the retina-SCN pathway by aging and AD progression. Statistical analysis was conducted by two-way ANOVA, followed by Tukey’s post hoc test. *** *p* < 0.001 and ^###^ *p* < 0.001 indicate a significant difference.

It is well-known that the RGCs project to the SCN of the hypothalamus [38]. In order to visualize the neural connections from the retina to the SCN, we injected CTβ into the vitreous of 4- and 12-month-old WT and 5XFAD mice. CTβ-positive signals were detected in the SCN of all the mouse groups (Figure 3A,B). To examine whether the retina-SCN pathway is altered in Aβ-overexpressing brains, we compared the fluorescence signals of CTβ in the SCN of the 5XFAD and WT mice. The 5XFAD group showed reduced CTβ fluorescence intensity in the SCN compared to the WT group [genotype: *F*(1,109) = 47.2, *p* < 0.0001], and the genotype difference was greater at 4 months than at 12 months. In addition, the fluorescence intensity of CTβ decreased with age [age: *F*(1,109) = 44.9, *p* < 0.0001], and the decrease was greater in the WT group than in the 5XFAD group. Moreover, a significant interaction between genotype and age was observed [interaction: *F*(1,109) = 23.84, *p* < 0.0001] (Figure 3C,D). Our findings show that no further impairment of the retina-SCN pathway is seen between 4- and 12-month-old 5XFAD mice since the impairment of the retina-SCN connection is thought to already reach a threshold in 4-month-old 5XFAD mice. Additionally, connectivity from the retina to the SCN is vulnerable to damage by normal aging. Consequently, these results suggest that the efferent projection from the retina to the SCN is seriously impaired before the prodromal stage of AD and is also impaired by the normal aging process.

### 3.3. Tracing the Retina-OPN Pathways in the 5XFAD Mice

**Figure 4:**
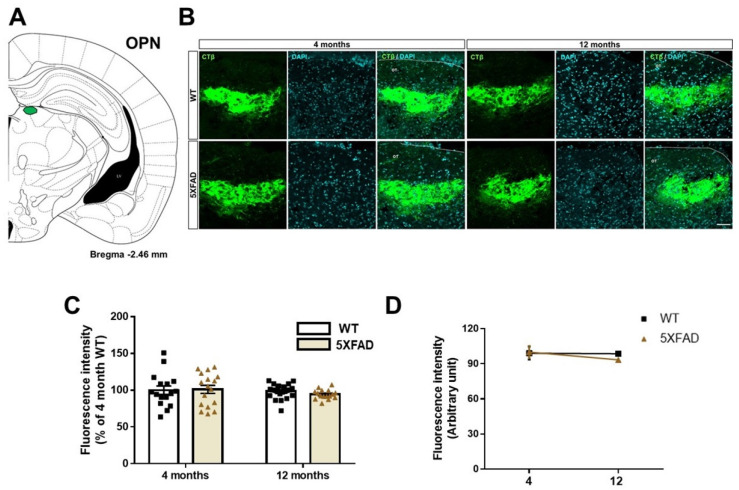
Anatomical tracing of the retina-olivary pretectal nucleus (OPN) pathways in WT and 5XFAD mice. (**A**) Diagram of a mouse brain section illustrating the location of the OPN. (**B**) The representative images show CTβ transported from the retina to the OPN. Scale bar = 50 μm. (**C**) The quantitative graphs exhibit the fluorescence intensity of the CTβ (+) areas in the OPN of the WT and 5XFAD mice (16−24 images of the brain sections were obtained from 5 mice in each group). (**D**) Comparison of the impairment of the retina-OPN projection by aging and AD progression. Statistical analysis was conducted by two-way ANOVA, followed by Tukey’s post hoc test.

To investigate whether the retinal outputs to OPN are altered in Aβ-overexpressing brains, we traced the transport of the anterograde tracer from the retina to the OPN in the 4- and 12-month-old WT and 5XFAD mice (Figure 4A,B). There was no difference in the fluorescence intensity of CTβ between WT and 5XFAD mice [genotype: *F*(1,69) = 0.1837, *p* = 0.6695]. Similarly, there was no difference of fluorescence intensity of CTβ between 4 months and 12 months groups [age: *F*(1,69) = 1.019, *p* = 0.3162]. Moreover, the retina-OPN connectivity did not show genotype × age interaction [interaction: *F*(1,69) = 0.5011, *p* = 0.4814] (Figure 4C,D). Our findings demonstrate that the retina-OPN pathway is rarely altered by AD progression and normal aging.

### 3.4. Decreased Retina-LGN Connectivity in the 5XFAD Mice

**Figure 5:**
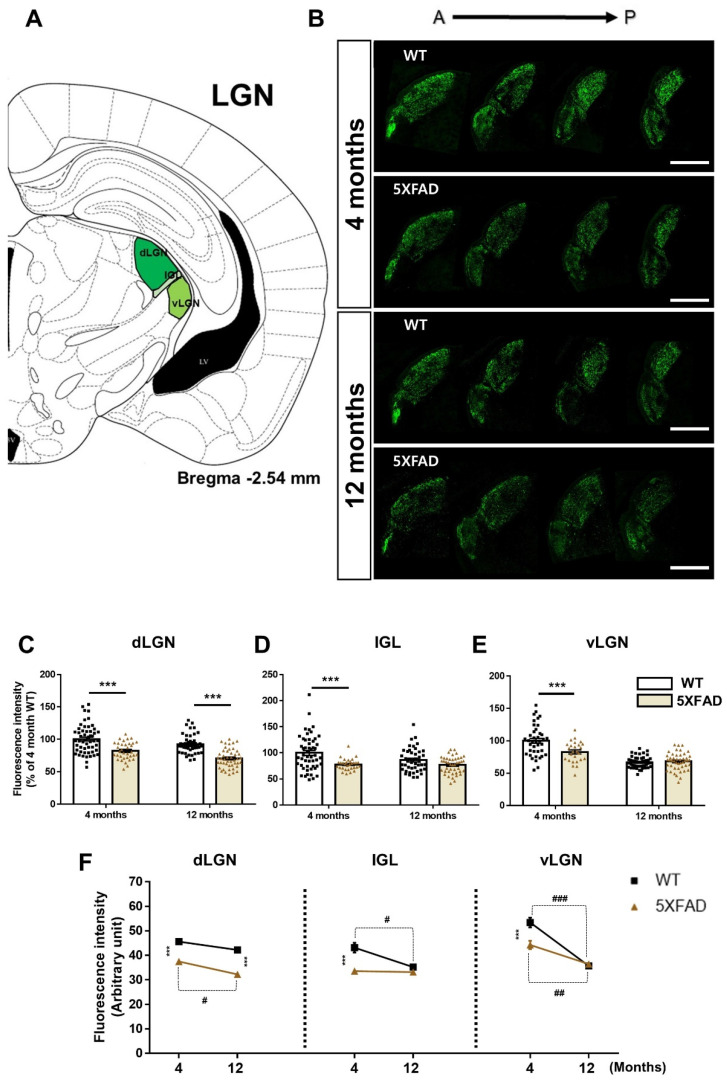
Changes of the retina-lateral geniculate nucleus (LGN) connections in WT and 5XFAD mice. (**A**) Image of a mouse brain atlas exhibiting the location of the LGN. (**B**) The representative images show CTβ transported from the retina to the LGN. Scale bar = 500 μm. The quantitative graphs exhibit the fluorescence intensity of CTβ (+) areas in the (**C**) dorsal lateral geniculate nucleus (dLGN), (**D**) intergeniculate leaflet (IGL), and the (**E**) ventral lateral geniculate nucleus (vLGN) of WT and 5XFAD mice (31−53 images of the brain sections were obtained from 5 mice in each group). (**F**) Comparison of the impairment of the retina-LGN pathways by aging and AD progression. Statistical analysis was conducted by two-way ANOVA, followed by Tukey’s post hoc test. *** *p* < 0.001, ^#^ *p* < 0.05, ^##^ *p* < 0.01, and ^###^ *p* < 0.001 indicate a significant difference.

The LGN is a crucial region in the visual pathway that relays visual information from the retina to the primary visual cortex [39]. We analyzed CTβ-positive signals in the three subregions of LGN, such as dLGN, IGL, and vLGN, in 4- and 12-month-old WT and 5XFAD groups (Figure 5A,B). First, the 5XFAD group showed decreased CTβ-positive signals compared to the WT group in the dLGN [genotype: *F*(1,177) = 62.58, *p* < 0.0001], and Tukey’s post hoc analysis showed that CTβ-positive signals were markedly reduced in 4- or 12-month-old 5XFAD mice than in age-matched WT mice. There was also an age-dependent reduction of CTβ-positive signals in the dLGN [age: *F*(1,177) = 14.69, *p* = 0.0002], and Tukey’s post hoc test indicated that CTβ-positive signals were considerably decreased in 12-month-old 5XFAD mice than in 4-month-old 5XFAD. There was no significant interaction between genotype and age in the dLGN [interaction: *F*(1,177) = 0.5611, *p* = 0.4548] (Figure 5C,F). The finding shows that the retinal projection to dLGN is more impaired by AD pathology than by aging.

Next, there was a significant difference in CTβ fluorescence intensity values between the WT and 5XFAD groups in the IGL [genotype: *F*(1,166) = 18.08, *p* < 0.0001], and Tukey’s post hoc analysis exhibited that CTβ fluorescence intensity was strikingly reduced in 4-month-old 5XFAD mice than in 4-month-old WT mice. The fluorescence intensity of CTβ decreased with age in the IGL [age: *F*(1,166) = 3.965, *p* = 0.0481], and Tukey’s post hoc test displayed that CTβ fluorescence intensity was significantly decreased in 12-month-old WT mice than in 4-month-old WT. There was no significant interaction between genotype and age in the IGL [interaction: *F*(1,166) = 2.973, *p* = 0.0865] (Figure 5D,F). Similar to the results of SCN, retinal to IGL projection was obviously impaired by AD pathology in 4-month-old 5XFAD mice. Additionally, impairments of this connection due to the normal aging process are prominent.

Finally, a significant difference in CTβ-labeled signals was found in the vLGN of WT and 5XFAD groups [genotype: *F*(1,160) = 9.364, *p* = 0.0026], and Tukey’s post hoc analysis showed that CTβ-labeled signals were significantly reduced in 4-month-old 5XFAD mice compared to 4-month-old WT mice. The CTβ-labeled signals decreased with age in the vLGN [age: *F*(1,160) = 86.99, *p* < 0.0001], and Tukey’s post hoc test presented that CTβ-labeled signals were significantly reduced in 12-month-old WT and 5XFAD mice than in 4-month-old WT and 5XFAD. There was significant interaction between genotype and age in the vLGN [interaction: *F*(1,160) = 13.13, *p* = 0.0004] (Figure 5E,F). The result reveals that retinal efferent projecting to the vLGN showed significance according to genotype in the early stage but showed significance according to genotype × age interaction in the later stage. Taken together, our findings demonstrate that (1) the retinal efferents to the dLGN, IGL, vLGN are severely damaged before the prodromal stage of AD, (2) the retina-dLGN and vLGN pathways are consistently impaired between prodromal and severe stages, and (3) the retinal projections to the IGL and vLGN changed during normal aging.

### 3.5. Altered Retina-SC Connection in the Aβ-Overexpressing Mice

**Figure 6:**
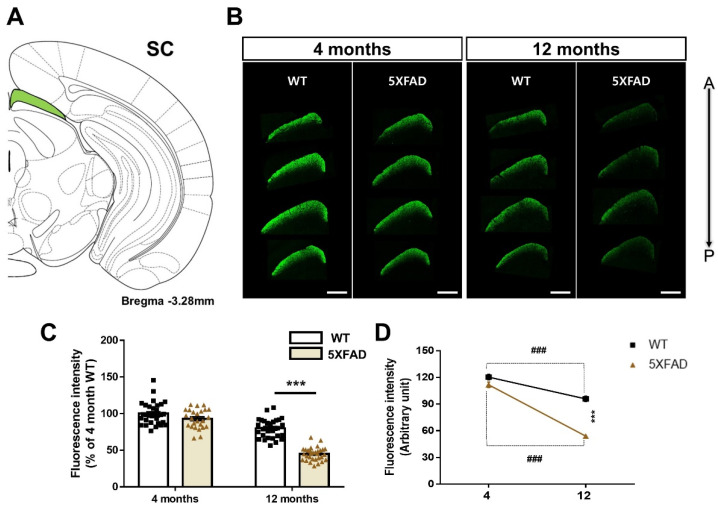
Degeneration of the retina-superior colliculus (SC) pathways in WT and 5XFAD mice. (**A**) Image of a mouse brain atlas illustrating the location of the SC. (**B**) The representative images indicate CTβ transported from the retina to the SC. Scale bar = 500 μm. (**C**) The quantitative graphs exhibit the fluorescence intensity of CTβ (+) areas in the SC of WT and 5XFAD mice (25–32 images of the brain sections were obtained from 5 mice in each group). (**D**) Comparison of the impairment of the retina-SC connection by aging and AD progression. Statistical analysis was conducted by two-way ANOVA, followed by Tukey’s post hoc test. *** *p* < 0.001 and ^###^ *p* < 0.001 indicate a significant difference.

The SC is a region that relays visual information as part of the extrageniculate visual pathway from the retina to the higher visual cortex and plays an important role in visual function [40]. To reveal the retina-SC pathway, we conducted anterograde-tracing with CTβ in 4- and 12-month-old WT and 5XFAD mice (Figure 6A,B). There was no impairment in the retina-SC pathway of 4-month-old 5XFAD mice compared to the 4-month-old WT mice. In contrast, the retina-SC connection of 12-month-old 5XFAD mice was significantly reduced compared to that of the 12-month-old WT mice [genotype: *F*(1,112) = 77.66, *p* < 0.0001]. Moreover, comparison of CTβ-positive axon terminals between 4-month-old and 12-month-old mice showed a significant age-related decrease in the retina-SC projection of both WT and 5XFAD mice [age: *F*(1,112) = 206, *p* < 0.0001]. A two-way ANOVA displayed a significant interaction between genotype and age [interaction: *F*(1,112) = 33.96, *p* < 0.0001], and Tukey’s post hoc analysis showed a more significant decrease in CTβ-positive axon terminals in 12-month-old 5XFAD mice than in WT groups or 4-month-old 5XFAD mice (Figure 6C,D). Therefore, these anterograde-tracing results demonstrate that the retina-SC projection is severely impaired between the prodromal and severe stages of AD and is also damaged by the normal aging process.

### 3.6. Decreased Retina-MTN Projections in the Animal Model of AD

**Figure 7:**
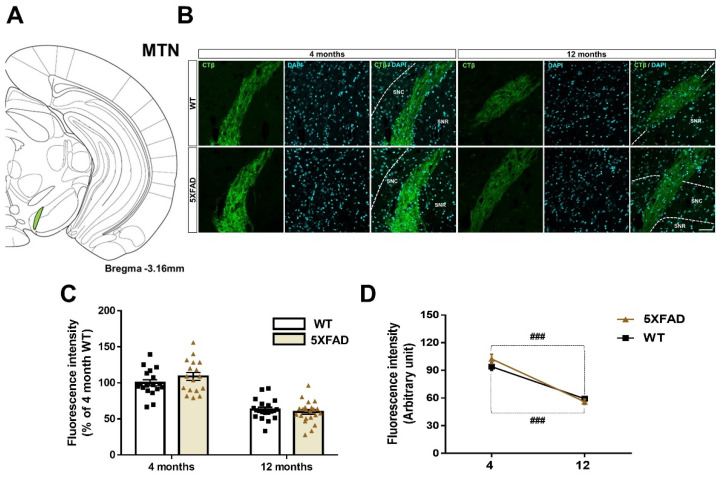
Neuroanatomic tract tracing of the retina-medial terminal nucleus of the accessory optic tract (MTN) projections in WT and 5XFAD mice. (**A**) Diagram of a mouse brain atlas illustrating the site of the MTN. (**B**) The representative images indicate CTβ transported from the retina to the MTN. Scale bar = 50 μm. (**C**) The quantitative graphs exhibit the fluorescence intensity of CTβ (+) areas in the MTN of WT and 5XFAD mice (18−22 images of the brain sections were obtained from 5 mice in each group). (**D**) Comparison of the impairment of the retina-MTN pathways by aging and AD progression. Statistical analysis was conducted by two-way ANOVA, followed by Tukey’s post hoc test. ^###^ *p* < 0.001 indicate a significant difference.

To reveal the changes in connectivity from the retina to the MTN in an AD mouse model, we detected the CTβ-positive signals in MTN of 4- and 12-month-old WT and 5XFAD groups (Figure 7A,B). CTβ-labeled signals were significantly reduced in 12 months of both WT and 5XFAD mice compared to 4 months [age: *F*(1,73) = 115.4, *p* < 0.0001] without a significant effect of genotype [genotype: *F*(1,73) = 0.4698, *p* = 0.4953] or a genotype × age interaction [interaction: *F*(1,73) = 2.369, *p* = 0.1281] (Figure 7C,D). Thus, the fluorescence intensity of CTβ in MTN changes only with age. These results suggest that retina-MTN pathways significantly degenerate during normal aging.

### 3.7. Neurodegeneration in the Retinorecipient Area in the 5XFAD Mice

**Figure 8:**
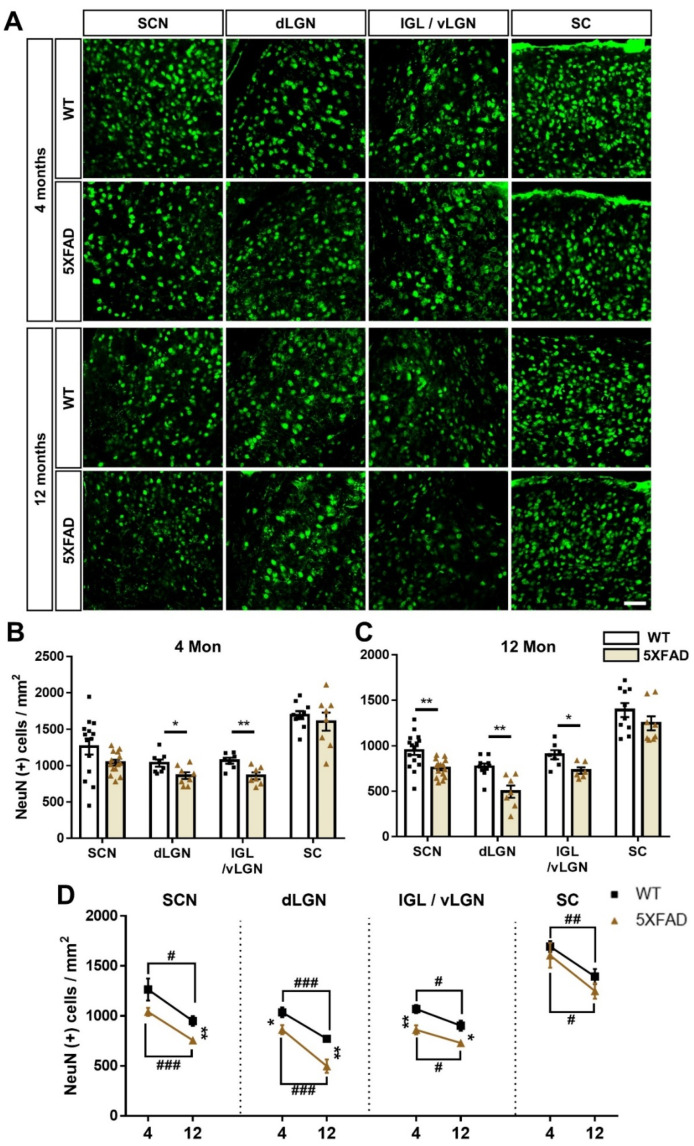
Neuronal loss in the retinorecipient area of the 5XFAD mice. (**A**) Representative images of neuronal nuclei (NeuN) in the SCN, dLGN, IGL, vLGN, and SC in the WT and 5XFAD mice. The scale bar is 50 μm. (**B**,**C**) The quantitative graphs exhibit the NeuN (+) cells in the SCN, dLGN, IGL, vLGN, and SC of WT and 5XFAD mice (6−16 images of the brain sections were obtained from 5 mice in each group). Statistical analysis was conducted by a multiple *t*-test. (**D**) Comparison of the neuronal cell death by aging and AD progression. Statistical analysis was conducted by two-way ANOVA, followed by Tukey’s post hoc test. * *p* < 0.05, ** *p* < 0.01, ^#^ *p* < 0.05, ^##^ *p* < 0.01, and ^###^ *p* < 0.001 indicate a significant difference.

**Figure 9:**
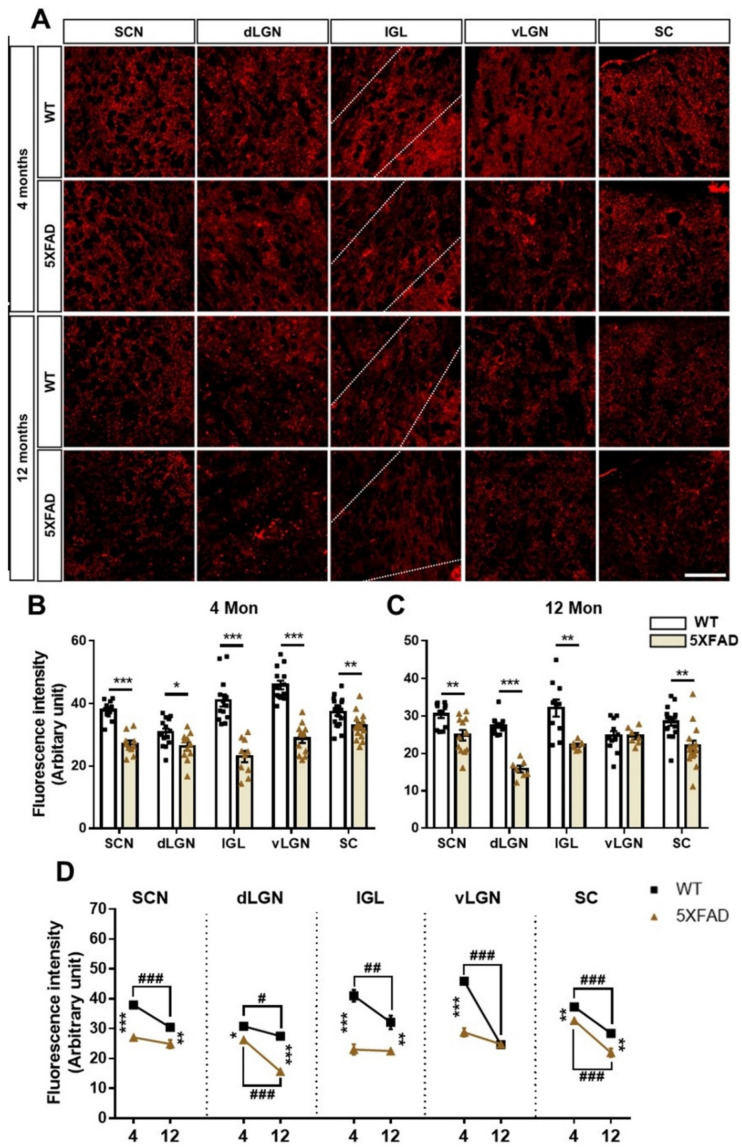
Synaptic degeneration in the retinorecipient area of the 5XFAD mice. (**A**) Representative images of pre-synaptic terminals in the SCN, dLGN, IGL, vLGN, and SC in the WT and 5XFAD mice. Pre-synaptic terminals were visualized using an anti-synaptophysin (SYN) antibody. The scale bar is 50 μm. (**B**,**C**) The quantitative graphs exhibit the fluorescence intensity of SYN (+) areas in the SCN, dLGN, IGL, vLGN, and SC of WT and 5XFAD mice (7−18 images of the brain sections were obtained from 5 mice in each group). Statistical analysis was conducted by a multiple *t*-test. (**D**) Comparison of the synaptic loss by aging and AD progression. Statistical analysis was conducted by two-way ANOVA, followed by Tukey’s post hoc test. * *p* < 0.05, ** *p* < 0.01, *** *p* < 0.001, ^#^ *p* < 0.05, ^##^ *p* < 0.01, and ^###^ *p* < 0.001 indicate a significant difference.

To investigate the cell death and synaptic degeneration in the exhibiting circuit damage from early AD stage, such as retina-SCN, retina-dLGN, retina-IGL, retina-vLGN, and retina-SC pathway, we conducted immunostaining to detect NeuN, a marker of neuronal nuclei (Figure 8A), and SYN, a marker of pre-synaptic terminals (Figure 9A). The 4-month-old 5XFAD mice indicated significant neuronal loss compared to WT mice in the dLGN, IGL, and vLGN (Figure 8B), which displayed robust accumulation of Aβ in 5XFAD mice. In 12-month-old 5XFAD mice, however, the SCN, dLGN, IGL, and vLGN regions exhibited significantly reduced numbers of NeuN-positive cells compared to WT mice; there was no significant reduce in the SC (Figure 8C). Interestingly, we found age- and AD progression-related neuronal loss in all regions (Figure 8D).

The SYN fluorescence intensity was significantly decreased in the SCN, dLGN, IGL, vLGN, and SC in 4-month-old 5XFAD mice (Figure 9B). The SCN, dLGN, IGL, and SC showed significant pre-synaptic degeneration in 12-month-old 5XFAD mice compared to WT mice (Figure 9C). Notably, we observed age-related pre-synaptic loss in all regions (Figure 9D). In particular, the pre-synaptic loss occurring in the SCN, dLGN, IGL, vLGN, and SC could be closely related to the decrease in the CTβ-positive signals in these regions. This finding could support a decrease in CTβ-positive signals in the brain areas without Aβ deposits. These results reveal that the neuronal and synaptic losses occur with aging and AD progression and that the neurodegeneration could significantly affect the impairment of neural circuits in the brain with AD.

## 4. Discussion

**Figure 10:**
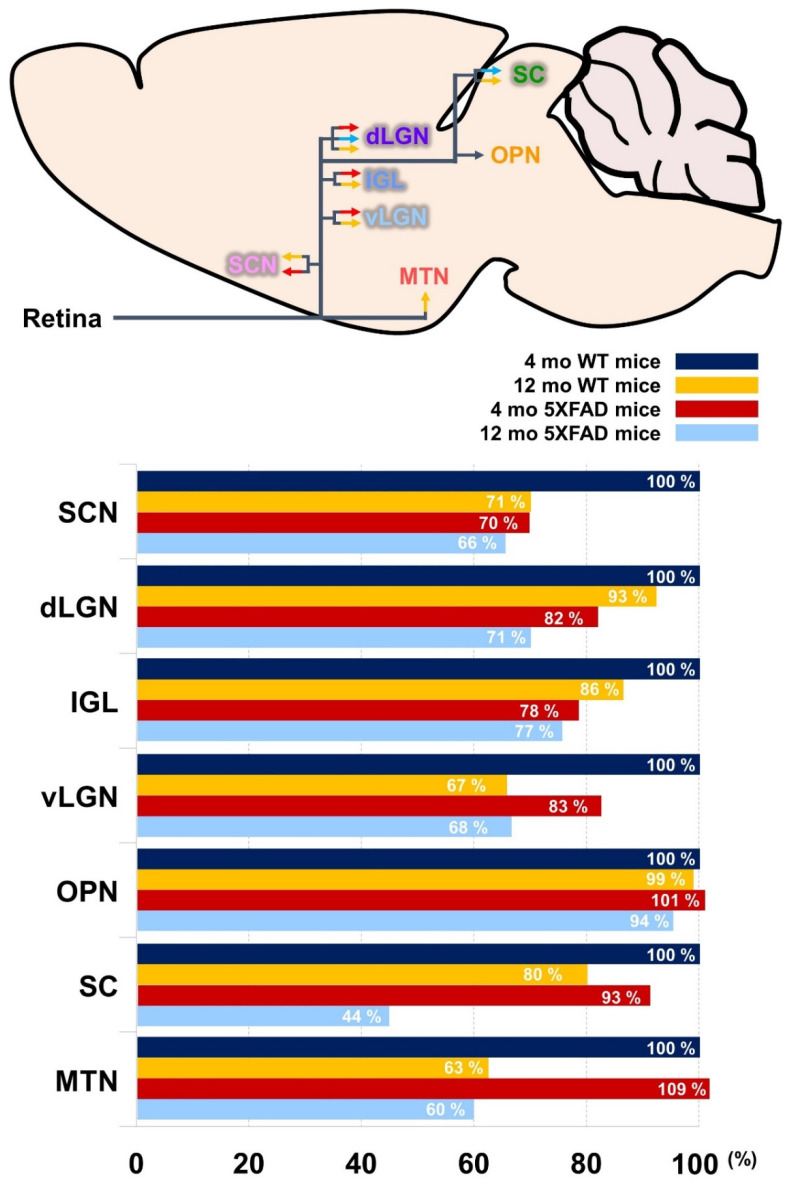
Schematic drawing and quantitative analysis of the altered visual pathways from the retina to the retinorecipient regions. The rate of degeneration of the visual pathways was normalized to those of 4-month-old WT mice indicated by the black bars. Red bars displayed regions that are impaired in early stage of AD. Blue bars indicate the damaged regions in late-stage of AD. Orange bars show regions damaged by aging.

Although visual circuit impairments in patients with AD patients has been reported at the macroscopic level [41], to the best of our knowledge, there is no study on the alteration of the visual circuit at the mesoscale level in the AD brain. Thus, our study aimed to provide direct anatomical evidence in a mouse model of AD by examining the topographical changes in various visual pathways between two different stages of AD. To investigate the visual circuitry of the AD brain, we intravitreally injected a non-transsynaptic anterograde trace CTβ into the right eye of 4- and 12-month-old WT and 5XFAD mice. We demonstrated that the retinal projections to the SCN, dLGN, IGL, and vLGN were impaired in the 4-month-old 5XFAD mice, which exhibit no cognitive decline, compared to the age-matched WT. In addition, we found decreased connections of retina-dLGN and retina-SC in the 12-month-old 5XFAD mice, representing the severe stage of AD, compared to age-matched WT. Finally, we revealed that retinal connectivity decreases in several retinorecipient areas except for the OPN by normal aging. Furthermore, we found that neuronal loss and synaptic degeneration occur in retinorecipient regions with aging and AD progression and that this neurodegeneration could significantly affect the impairment of neural circuits. The present study demonstrated for the first time, the alteration of the visual pathways from the retina in the AD brain at the mesoscale level (Figure 10).

Retinal connections to the SCN, vLGN, and IGL were significantly impaired in the 4-month-old 5XFAD mice. However, these pathways did not show significant changes in CTβ-positive signals between 4 and 12 months of 5XFAD (Figure 3D and Figure 5F). One of the reasons is that these visual circuits in 5XFAD mice began to be impaired early, reaching thresholds already in 4-month-old 5XFAD mice. Neural circuit impairment that has reached a threshold result in early visual-related behavioral impairments. Many studies reported that the SCN, vLGN, and IGL are regions partially involved in circadian rhythms, such as the sleep-wake cycle [42,43]. Interestingly, circadian rhythm disturbance was observed in 4-month-old 5XFAD mice [23,44]. Thus, this evidence suggests that retinal projections to the SCN, vLGN, and IGL are already sufficiently impaired in 4-month-old 5XFAD mice, suggesting that alterations in these circuits may in part contribute to abnormal circadian rhythms.

Retinal projection to dLGN is consistently decreased in 4- and 12-month-old 5XFAD mice compared to WT of the same age (Figure 5F). These results demonstrate that the retina-dLGN pathway is continuously damaged during AD progression. Unfortunately, few studies have reported visual impairments, such as contrast sensitivity and color vision, in the 5XFAD mouse model. However, a study using an APP^SWE^/PS1^∆E9^ mouse model reported that contrast sensitivity and color vision were impaired in AD progression [45]. This finding indicates that the retinal-dLGN pathway is persistently impaired in AD progression and damage to this circuitry can result in loss of contrast sensitivity and color vision.

The retina-SC pathway was dramatically reduced in the 12-month-old 5XFAD mice. However, this pathway did not significant changes in CTβ-positive signals between 4 months of WT and 5XFAD mice (Figure 6D). The result suggests that damage to this pathway in 5XFAD mice begins at some point between 4 and 12 months of age. Some studies show that the SC is involved in visuomotor functions, such as eye movement [46,47]. Visual acuity (optokinetic system) impairment was observed in 5XFAD mice from 6 months of age [22]. Thus, this evidence suggests that the connection between the retina and SC is significantly impaired between 4- and 12-month-old 5XFAD mice, suggesting that impairment of these circuits may in part contribute to visuomotor impairment.

It is well known that Aβ-induced neuronal dysfunctions induce neuronal circuit and network disturbances [48]. A key component of these neuronal dysfunctions is axonal degeneration. Axonal degeneration by Aβ toxicity preceded neuronal cell death. In contrast, inhibition of axonal degeneration through overexpression of nicotinamide mononucleotide adenylyltransferase 1 and Bcl-xl prevented neuronal cell death [49]. Therefore, Aβ observed in the retina (Figure 1E,F) can cause axonal degeneration of RGCs, resulting in disruption of the visual pathway. Similarly, the neuronal and synaptic loss occurred in the SCN, dLGN, IGL, vLGN, and SC of 4-month-old 5XFAD mice with/without Aβ deposition in those regions. These results could be due to already initiated neurodegeneration by intraneuronal Aβ-induced neurotoxicity, which appears much earlier than Aβ deposition [30,50].

It has been reported that 5XFAD mice, an Aβ overexpressing mouse model, exhibited similar levels of Aβ_40_ and Aβ_42_ peptides to those in the retina and neocortex of AD patients [51]. However, further experiments are needed to characterize the AD stage-dependent Aβ aggregates form in 5XFAD mouse retinas, as well as studies of retinal Aβ accumulation patterns in AD animal models and AD patients. Nevertheless, the 5XFAD mice showed stronger retinal and synaptic pathology than other AD mouse models [51]. Significantly increased Aβ concentrations were observed in the eyes of young 5XFAD mice without cognitive impairment compared to WT mice of the same age [18,52,53]. In addition, visual behavior abnormalities and circadian rhythm disturbance were reported in 5XFAD mice [18,22,23,44,54]. Although 5XFAD mouse models do not reflect all of the pathologies of AD and may not reflect the same visual pathway impairment as patients with AD, this model recapitulates several aspects, such as structural, molecular, cellular, and behavioral phenotypes, observed in AD patients. For instance, neuronal cell death and synaptic loss induced by Aβ are strong correlates of cognitive deficits in AD. Likewise, neurodegeneration induced by Aβ in 5XFAD mice is temporally associated with various dysfunctions. Therefore, since non-clinical studies are needed for developing treatment and diagnostic methods for AD, among various AD models, we chose 5XFAD mice, which accumulate the Aβ in the retina at the early stage and exhibit visual-related symptoms.

Our results suggested the retinal outputs were impaired in the 5XFAD mice. Unfortunately, most conventional tracers are unable to reveal the connectivity at the level of the cell-type-specific pathway [55]. Therefore, genetic tracing using cell-type-specific promoters should be used to investigate the disruption of glutamate-specific visual pathways. In addition, further studies on retinal projection damaged by hyperphosphorylated tau in tau animal models are needed, as phosphorylated tau may affect neural circuitry. Moreover, further studies using 5XFAD mice of various ages are needed to identify points where damage to the visual circuit is prominent. Nevertheless, the current research is the first to demonstrate the alteration in the visual pathways in an animal model of AD at the mesoscale level. Restoring damaged visual pathways with optogenetic stimulation, based on mesoscopic mapping of connectivity in the AD brain, could be a possible therapeutic approach for treating visual symptoms in patients with AD.

## 5. Conclusions

In this study, we examined the alteration of the visual pathway in the animal model of AD at the mesoscale level in both the early and late stages of AD. Taken together, the results of our study using 5XFAD mice demonstrated that (1) retinal projections to the SCN, dLGN, IGL, and vLGN were significantly impaired in the early stages of AD, (2) connections of the retina with dLGN and SC were disrupted in the late stages of AD, (3) the visual pathways in the brain with AD showed a trend towards accelerated degeneration during the AD progression, and (4) the visual circuits in the healthy brain degenerated according to aging.

## Data Availability

Not applicable.

## Supplementary Materials

The following supporting information can be downloaded at: https://www.mdpi.com/article/10.3390/cells11233901/s1, Figure S1. Preparation of brain tissues of mice for CTβ-positive signals and immunoreactivity analysis. Fixed mouse brains were coronally sectioned with a thickness of 30 μm. From 1 to 2, the suprachiasmatic nucleus (SCN) is located from −0.22 mm to −0.82 mm of the bregma and is labeled in blue. From 3 to 4, the olivary pretectal nucleus (OPN) is located from −2.18 mm to −2.46 mm of the bregma and is labeled in violet. From 3 to 5, the lateral geniculate nucleus (LGN) is located from −2.18 mm to −2.92 mm of the bregma. The LGN subregions, the dorsal lateral geniculate nucleus (dLGN), intergeniculate leaflet (IGL), and ventral lateral geniculate nucleus (vLGN), are shown in yellow, orange, and green, respectively. From 6 to 7, the medial terminal nucleus of the accessory optic tract (MTN) is located from −3.16 mm to −3.28 mm of the bregma and is labeled in magenta. From 6 to 8, the superior colliculus (SC) is located from −3.16 mm to −4.36 mm of the bregma and is labeled in red. For quantification of CTβ-positive signals, we obtained seven to eight sections per mouse in each region. For quantification of immunoreactivity, we acquired four sections per mouse in each region. The received images were subjected to topographical quantification and statistical analysis in a blind manner; Figure S2. The topographical schematic diagram for quantifying CTβ-positive signals and immunoreactivity analysis in the brain tissues. (A) We show the process of the analysis steps for “area fraction (%)”, “fluorescence intensity (Arbitrary units)” and “(+) cells/mm^2^” using the ImageJ program. It displays the quantitative process of “Area fraction (%)” applied to histological analysis of 4G8-positive signals. (C) It shows the quantitative process of “Fluorescence intensity (Arbitrary units)” applied to histological analysis of CTβ and SYN-positive signals. (D) It exhibits the quantitative process of “(+) cells/mm^2^” applied to histological analysis of NeuN (+) cells; Figure S3. Representative image of the photomicrograph validation of the intravitreal injection sites of the CTβ in the retinal flat mount. Scale bar = 500 μm; Figure S4. Representative images are immunohistochemical staining using anti-4G8 antibody in WT mice. Scale bar = 50 μm; Figure S5. Validation of the application of the non-transsynaptic anterograde tracer CTβ in the suprachiasmatic nucleus (SCN). (A) Representative images show the co-localization of CTβ with synaptophysin (SYN), marker of presynaptic terminals. Scale bar = 10 μm. (B) Profile analysis of representative images shows that CTβ is predominantly co-localized with SYN. The graph shows the fluorescence intensity of the white dotted line. White arrows indicate where CTβ (Green) and SYN (Red) coexist.
