# Supplementary material for: Mesoscopic Mapping of Visual Pathway in a Female 5XFAD Mouse Model of Alzheimer’s Disease
Source: Cells. 2022 Dec 2;11(23):3901. doi: 10.3390/cells11233901 (PMC9740259; doi:10.3390/cells11233901)
Supplement: Supplementary file 1 [file cells-11-03901-s001.zip › cells-1957008-supplementary.pdf]

Supplementary Figures

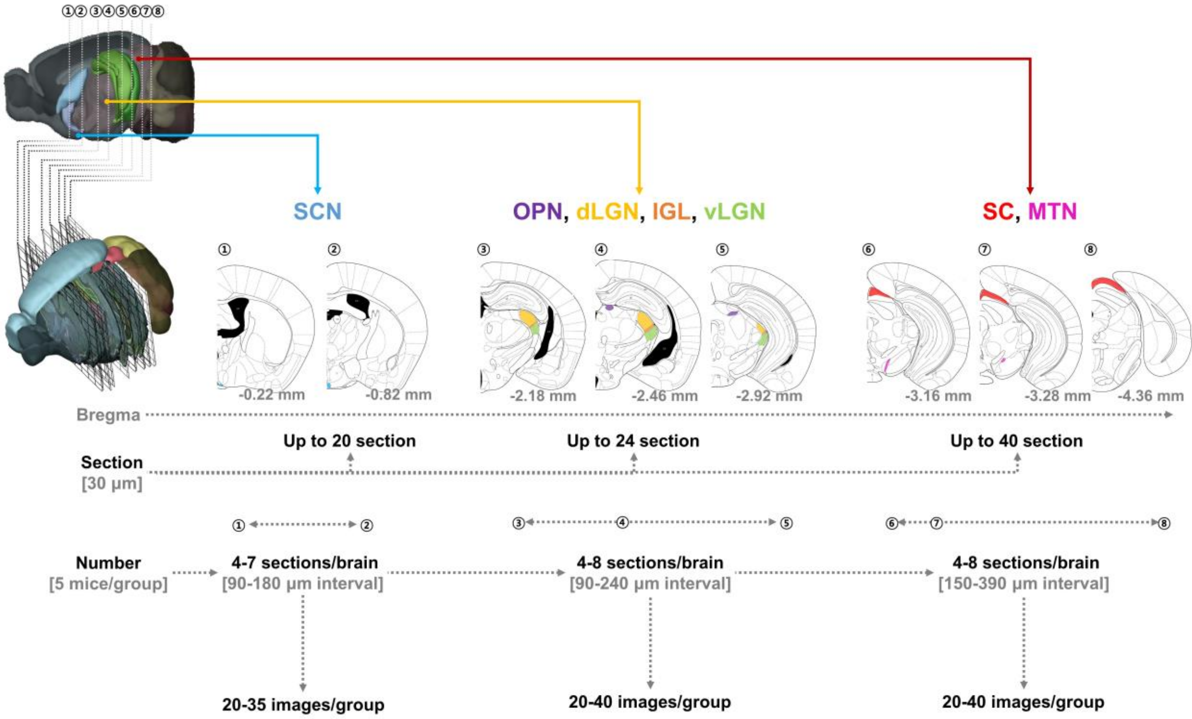

Supplementary Figure S1. Preparation of brain tissues of mice for CTβ-positive signals and immunoreactivity analysis. Fixed mouse brains were coronally sectioned with a thickness of 30 μm. From 1 to 2, the suprachiasmatic nucleus (SCN) is located from -0.22 mm to -0.82 mm of the bregma and is labeled in blue. From 3 to 4, the olivary pretectal nucleus (OPN) is located from -2.18 mm to -2.46 mm of the bregma and is labeled in violet. From 3 to 5, the lateral geniculate nucleus (LGN) is located from -2.18 mm to -2.92 mm of the bregma. The LGN subregions, the dorsal lateral geniculate nucleus (dLGN), intergeniculate leaflet (IGL), and ventral lateral geniculate nucleus (vLGN), are shown in yellow, orange, and green, respectively. From 6 to 7, the medial terminal nucleus of the accessory optic tract (MTN) is located from -3.16 mm to -3.28 mm of the bregma and is labeled in magenta. From 6 to 8, the superior colliculus (SC) is located from -3.16 mm to -4.36 mm of the bregma and is labeled in red. For quantification of CTβ-positive signals, we obtained seven to eight sections per mouse in each region. For quantification of immunoreactivity, we acquired four sections per mouse in each region. The received images were subjected to topographical quantification and statistical analysis in a blind manner.

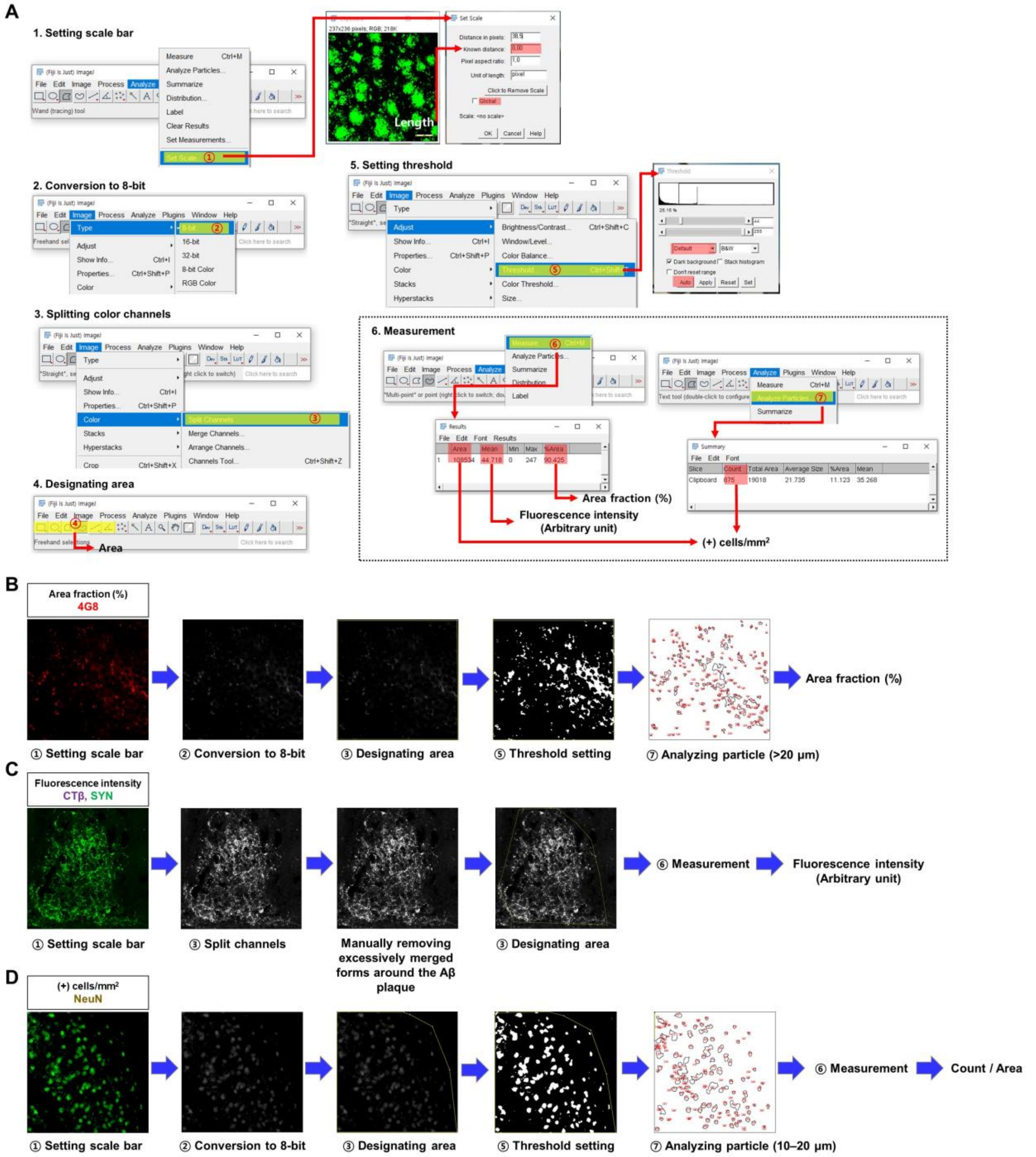

Supplementary Figure S2. The topographical schematic diagram for quantifying CTβ-positive signals and immunoreactivity analysis in the brain tissues. (A) We show the process of the analysis steps for "area fraction (%)", "fluorescence intensity (Arbitrary units)" and "(+) cells/mm^2" using the ImageJ program. It displays the quantitative process of "Area fraction (%)" applied to histological analysis of 4G8-positive signals. (C) It shows the quantitative process of "Fluorescence intensity (Arbitrary units)" applied to histological analysis of CTβ and SYN-positive signals. (D) It exhibits the quantitative process of " (+) cells/mm2" applied to histological analysis of NeuN (+) cells.

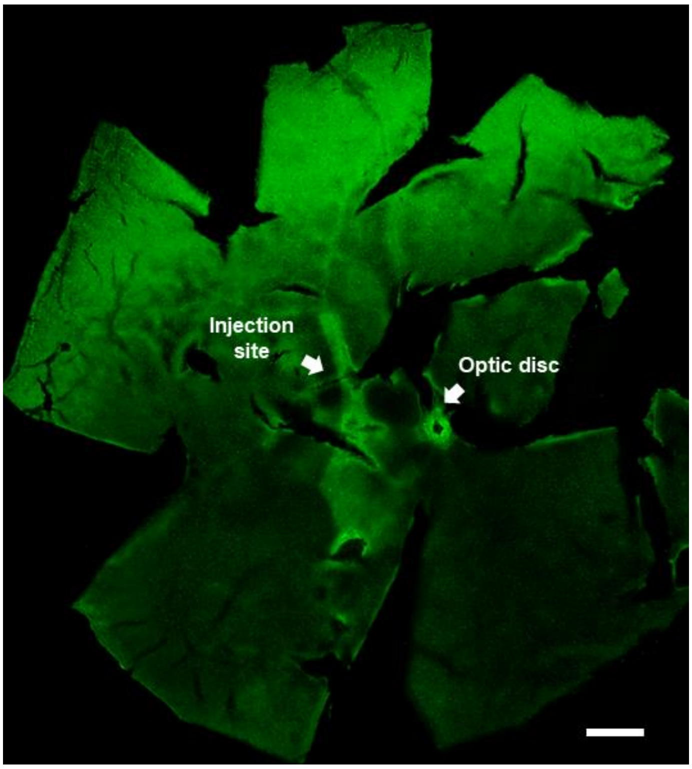

Supplementary Figure S3. Representative image of the photomicrograph validation of the intravitreal injection sites of the CTβ in the retinal flat mount. Scale bar = 500 μm.

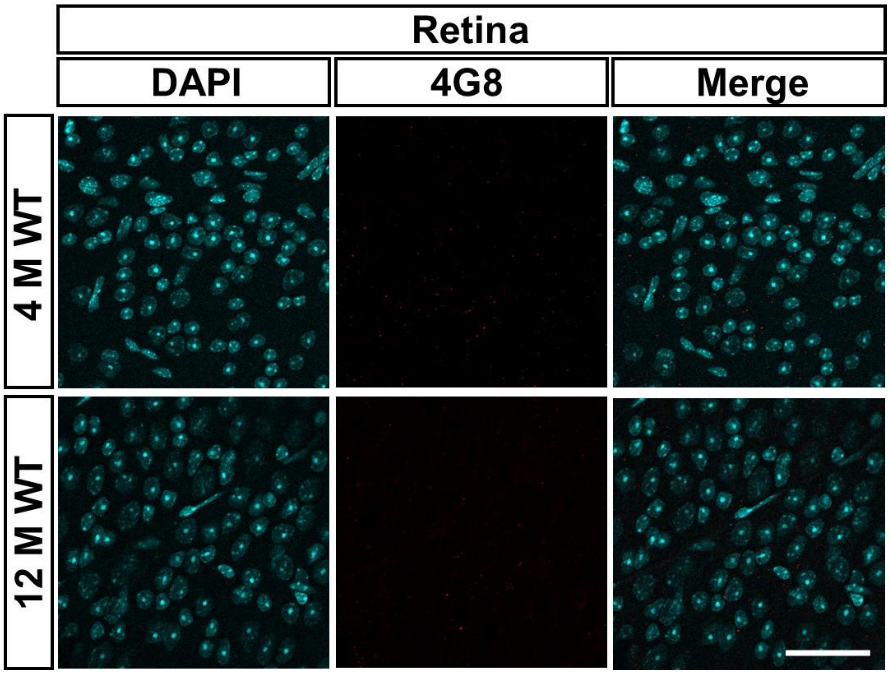

Supplementary Figure S4. Representative images are immunohistochemical staining using anti-4G8 antibody in WT mice. Scale bar = 50 μm.

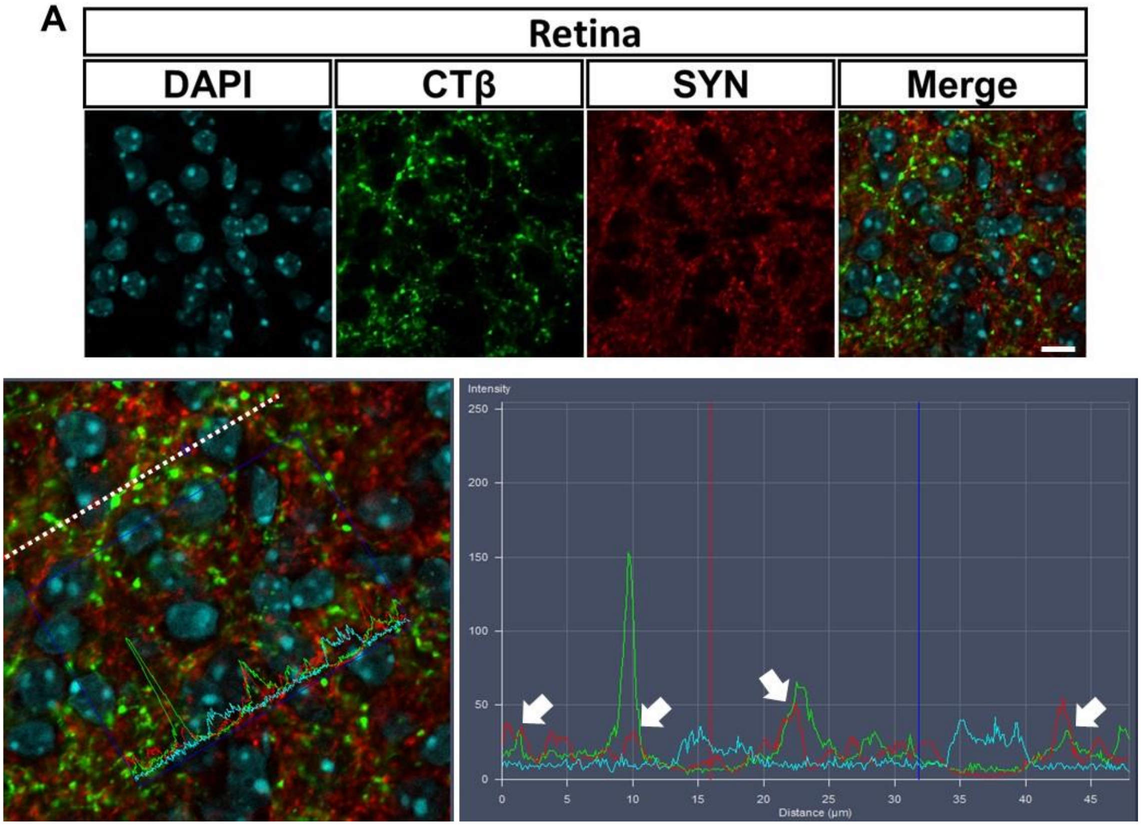

Supplementary Figure S5. Validation of the application of the non-transsynaptic anterograde tracer CTβ in the suprachiasmatic nucleus (SCN). (A) Representative images show the co-localization of CTβ with synaptophysin (SYN), marker of presynaptic terminals. Scale bar = 10 μm. (B) Profile analysis of representative images shows that CTβ is predominantly co-localized with SYN. The graph shows the fluorescence intensity of the white dotted line. White arrows indicate where CTβ (Green) and SYN (Red) coexist.
